# Slurry Injection Schemes on the Extent of Slurry Mixing and Availability during Chemical Mechanical Planarization

**DOI:** 10.3390/mi8060170

**Published:** 2017-05-29

**Authors:** Matthew Bahr, Yasa Sampurno, Ruochen Han, Ara Philipossian

**Affiliations:** 1 Department of Chemical and Environmental Engineering, University of Arizona, Tucson, AZ 85721, USA; yasayap@email.arizona.edu (Y.S.); hanr@email.arizona.edu (R.H.); ara@email.arizona.edu (A.P.); 2 Araca, Inc., Tucson, AZ 85718, USA

**Keywords:** slurry availability, slurry injection system, slurry injection position, chemical mechanical planarization, CMP, slurry utilization efficiency

## Abstract

In this study, slurry availability and the extent of the slurry mixing (i.e., among fresh slurry, spent slurry, and residual rinse-water) were varied via three different injection schemes. An ultraviolet enhanced fluorescence technique was employed to qualitatively indicate slurry availability and its flow on the pad during polishing. This study investigated standard pad center area slurry application and a slurry injection system (SIS) that covered only the outer half of the wafer track. Results indicated that the radial position of slurry injection and the alteration of fluid mechanics by the SIS played important roles in slurry mixing characteristics and availability atop the pad. Removal rates were found to decrease with slurry availability, while a higher degree of slurry mixing decreased the fraction of fresh slurry and consequently lowered the removal rate. By using a hybrid system (i.e., a combination of slurry injection via SIS and standard pad center slurry application), the polishing process benefited from higher slurry availability and higher fraction of fresh slurry than the conventional pad center slurry application and the shorter SIS, individually. This work underscores the importance of optimum slurry injection geometry and flow for obtaining a more cost-effective and environmentally benign chemical mechanical planarization process.

## 1. Introduction

Chemical mechanical planarization (CMP) is an enabling step in integrated circuit (IC) manufacturing for achieving local and global surface planarity through combined chemical and mechanical means. CMP has been widely used in the semiconductor manufacturing industry since 1985 [1]. Previous planarization technologies such as thermal flow and spin-on glass have been shown to provide adequate local planarity, but CMP is the only technique that additionally provides global planarity across the wafer surface [2]. A lack of global surface planarity results in tremendous difficulties during the following steps of the IC manufacturing process, such as lower photolithography and etch yields, greater step height variation, greater line-width variation, and the amplification of previous layer defects [3]. These variations critically impact and reduce both chip performance and overall device yield [1]. In addition to planarity, CMP must address issues concerning the surface quality of the wafer. Many academic and industry studies have shown that by controlling the process parameters and consumable sets, average surface roughness values ranging from 0.5 nm to 2 nm can be achieved [3,4,5,6]. 

The CMP process requires several consumables, such as polishing pad, pad conditioner disc, retaining ring, and slurry. The CMP slurry contains chemicals and nano-particles to support the chemical and mechanical actions of the removal process. In most CMP processes, slurry cost is a major component (up to 50%) of the overall cost of ownership [7,8]. Furthermore, slurry consumption can have serious environmental ramifications, as spent slurries contain hazardous chemicals as well as significant amounts of abrasive nano-particles. Even though the recovery and reuse of spent slurries has been investigated and adopted by a handful of IC manufacturers [9,10], we believe that the best solution is to optimize slurry usage by either reducing its flow rate to attain the same CMP performance (i.e., removal rate and defects) or by applying the same flow rate to achieve better performance as compared to the current process-of-record.

**Figure 1:**
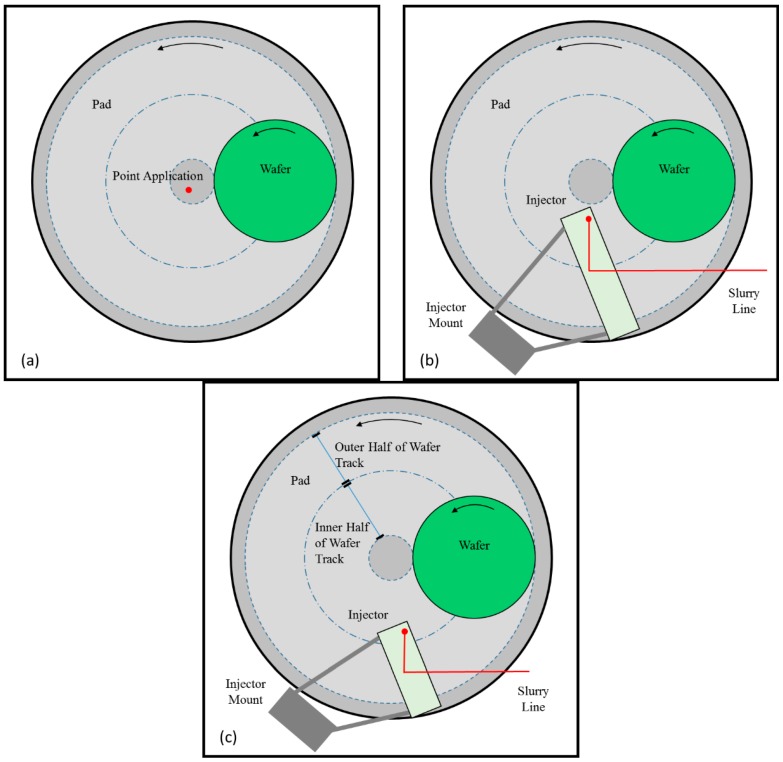
Top views of a polisher with (**a**) the standard slurry application method, (**b**) a slurry injection system (SIS) design that covers the whole wafer track, and (**c**) a SIS design that covers only the outside half of the wafer track.

In most commercially available polishers, slurry is applied near the center of the pad, as shown in Figure 1a. As the pad continuously rotates, a large amount of fresh slurry flows radially off the pad surface without ever entering the pad–wafer interface. Such a flow pattern results in extremely low slurry utilization efficiencies [8,11]. Several other methods have been proposed for applying or injecting slurry onto the pad surface. For example, Mok has proposed an apparatus for spraying slurry directly on to the pad surface rather than dispensing the slurry via the standard pad-center stream application [12]. Chamberlin et al. have proposed a similar slurry injection technique involving spraying pressurized slurry over the pad through multiple nozzles [13]. Chiou et al. have also proposed a modified slurry dispensing apparatus employing “a plurality of adjustable nozzles” [14]. Finally, Chang has proposed a method for dispensing slurry through multiple nozzles above the pad near the pad–wafer interface which tends to promote slurry coverage over the entire wafer track [15]. It must be noted that while these methods help deliver fresh slurry to the pad, none of them prevent the mixing of spent slurry and residual rinse water with fresh slurry during polishing.

Polishing processes continuously generate spent slurry on the pad surface. Spent slurries often contain pad debris (from the conditioning process and also from normal pad wear), diamond chips (that may get dislodged from diamond discs), and chemical by-products. Studies have shown that these contaminants can decrease material removal rate and increase wafer-level defects [11,16]. To mitigate such issues, large amounts of ultrapure water (UPW) are used to rinse the pad between polishes. Following pad rinsing, appreciable amounts of residual rinse water still reside on the land areas of the pad as well as inside the grooves. When fresh slurry is injected onto the pad during polishing, it mixes with the residual rinse water and gets diluted. As most industrially-relevant slurries result in lower removal rates when further diluted with water, over-the-pad mixing of water and slurry should be avoided. As such, it is fair to say that the current standard pad center slurry application method as well as the aforementioned slurry application or injection methods do not provide efficient slurry utilization. This has provided us with the opportunity to improve polishing performance. 

As an alternate method, Meled et al. and Mu et al. investigated a slurry injection system (SIS) which is aimed to shorten the slurry mean residence time (MRT) on the pad surface [11,17]. In both studies, the SIS was placed adjacent to the wafer on the pad surface covering the whole wafer track, as shown in Figure 1b. The SIS facilitated the delivery of fresh slurry to the pad–wafer interface. In addition, the SIS effectively blocked the spent slurry and residual rinse water from re-entering the pad–wafer interface and therefore allowed a higher fraction of the fresh slurry to be delivered to the polishing region. As a result, the SIS achieved a significantly lower slurry mean residence time, higher removal rate, and lower polishing defects than the standard pad center area slurry application method [11,17].

It must be emphasized that Meled et al. and Mu et al. employed an SIS design that covered the whole wafer track on the surface of the polishing pad as shown in Figure 1b. As a matter of a fact, some CMP systems designed for high volume manufacturing have particular space restrictions that will not allow the implementation of SIS units that cover the entire wafer track on the surface of the pad. Such space restrictions are typically associated with oscillations of the conditioner and the wafer carrier head. As a continuation of both Meled and Mu’s works, this study investigates an SIS design that covers only the outer half of the wafer track, as shown in Figure 1c. This study aims to understand if such an SIS unit—which may need to be shorter in length only because of possible space limitations on the pad—can provide similar benefits as the full-size SIS design. In addition, an ultraviolet enhanced fluorescence (UVEF) technique is employed to qualitatively measure the slurry availability on top of the pad prior to its entry into the pad–wafer interface. When compared to the standard pad center slurry application method, results help to confirm the main mechanism responsible for the enhanced removal rate associated with the SIS. 

## 2. Materials and Methods

The standard pad center area slurry application method and a novel method for slurry injection system were used to apply slurry onto the pad surface. Figure 1a,c show the top views of a polisher with the standard slurry application method and the SIS design that only covers the outer half of the wafer track. For the standard pad center area slurry application method (what we will henceforth refer to as “Point Application” or “PA”), the slurry is applied above the pad center. For the SIS, the system consists mainly of an injector and an injector mount. The injector has a rectangular shape which is attached to the injector mount with the connecting rods. The bottom of the injector is in contact with the surface of the polishing pad. The mount is used to securely attach the entire SIS assembly to the polisher’s frame. A single slurry inlet port is placed on top of the body which matches an outlet at its bottom y (at the trailing edge). Fresh slurry is introduced through the slurry feed line from the slurry tank where it flows into the inlet and then flows out into a channel machined into the bottom of the injector body. This channel helps to evenly spread the fresh slurry onto pad surface during polishing. A full description of the SIS can be found elsewhere [18]. In one test configuration, a hybrid slurry injection method is employed. The rationale behind this hybrid injection method is discussed in detail in Section 3 of this paper. 

All wafer polishing was done on an Araca APD-800X polisher. Detailed description of this polisher may be found elsewhere [19]. A 3M A165 diamond disc was used to perform in-situ conditioning on an IC-1000 pad (manufactured by Dow) with a “K-groove” pattern. The conditioning down-force was set to 44.5 N. Each wafer was polished for 1 min at 27.6 kPa and 1.5 m/s. Before polishing, pad break-in was performed with the diamond disc for 60 min with DI water. The conditioning disc’s rotation rate was set to 95 RPM, and its sweep frequency across the radius of the pad to 10 times per min. The diamond disc, pad, and wafer rotations were all counter clockwise. Pad break-in was then followed by pad seasoning, during which the shear force was monitored in real-time to ensure that stable values were achieved prior to any polishing with monitor wafers. It is important to note that even though we selected a relatively hard pad for the polishing tests, the application and utility of the SIS is by no means limited to hard pads.

The Semi-Sperse^®^ 25 slurry (manufactured by Cabot Microelectronics, Aurora, IL, USA)—diluted with water to a final solids content of 12.5% by weight—was used as the polishing slurry. Slurry flow rates were set at 150 and 250 mL/min. Blanket silicon dioxide wafers (300-mm) were polished for all injection schemes. Before and after polishing each wafer, a reflectometer from SENTECH Instruments GmbH (Berlin, Germany) was used to measure the thickness of the silicon dioxide film, which allowed us to compute the average removal rate for each test. Within-wafer removal rate non-uniformity (WIWRRNU) as well as wafer-level large particle counts were not determined in this study. However, in an earlier work by our team [20] using similar consumables and process conditions, SIS yielded equivalent values for WIWRRNU (3.9 ± 0.6% vs. 4.0 ± 0.5% for PA, 1-σ with 5 mm edge exclusion) and significantly lower wafer-level, greater than 0.5 micron, particle counts (174 ± 57 vs. 438 ± 155 for PA, 1-σ with 5 mm edge exclusion). Furthermore, it should be noted that our objective here was to polish silicon dioxide films, but the methods and techniques employed in our work are also applicable to other insulating (i.e., silicon nitride) as well as conducting (i.e., tungsten, tantalum, and copper) films.

**Figure 2:**
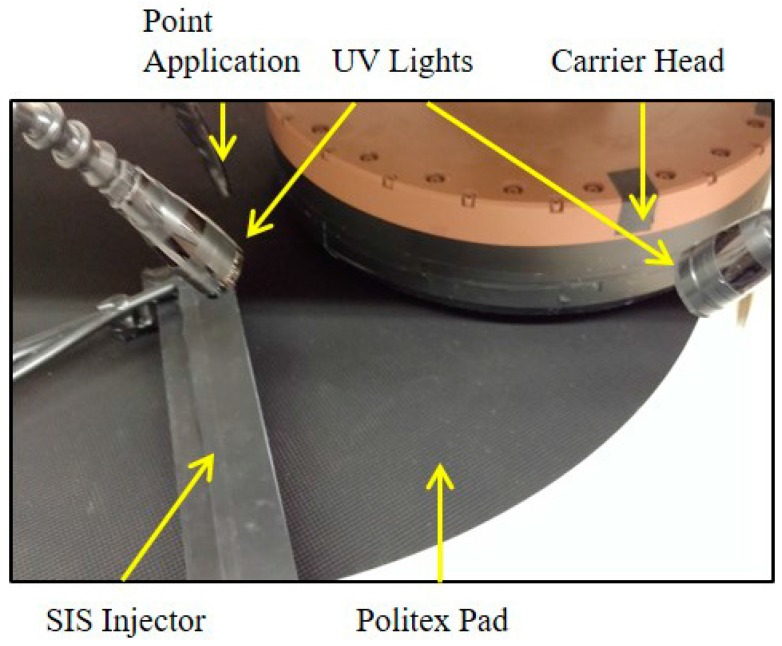
Experimental setup for ultraviolet enhanced fluorescence (UVEF) on an Araca APD-800 Polisher.

In addition to the above series of polishing tests, an ultraviolet enhanced fluorescence (UVEF) technique was employed to qualitatively visualize slurry flow patterns and measure the availability of the slurry (i.e., its thickness) on the pad surface [21,22]. Before taking images, an embossed Politex pad (manufactured by Dow Electronic Materials, Newark, DE, USA)—which is softer compared to IC-1000—was conditioned using a 3M PB32A brush for 30 min with UPW at a conditioning force of 13.3 N. The Politex pad was employed because of its black color, not its mechanical properties, as in UVEF tests attaining superior color contrast between the fluorescing dye and the pad is critical. In all experiments, the pad was conditioned in-situ. The slurry for the UVEF experiments consisted of 1 volume part of Fujimi PL-7103 slurry, 4 volume parts of DI water, and 0.5 g/L of 4-methyl-umbelliferone. Pad rinsing was performed in between polishes using UPW. The experimental setup is shown in Figure 2. Ultraviolet (UV) light from two light-emitting diodes was projected onto the leading edge of the carrier head. As the slurry was tagged with a fluorescent dye (i.e., 4-methyl-umbelliferone), the UV light excited the dye in the slurry, causing it to fluoresce. The intensity of the emitted fluorescence was proportionate to the amount of the slurry (i.e., the thickness of the slurry film) [21,22]. A high-resolution charged coupled device camera was employed to record the emission of fluorescent light on the leading edge of the wafer carrier head. The images were then analyzed via a customized software written in LabVIEW [21,22].

## 3. Results and Discussion

**Figure 3:**
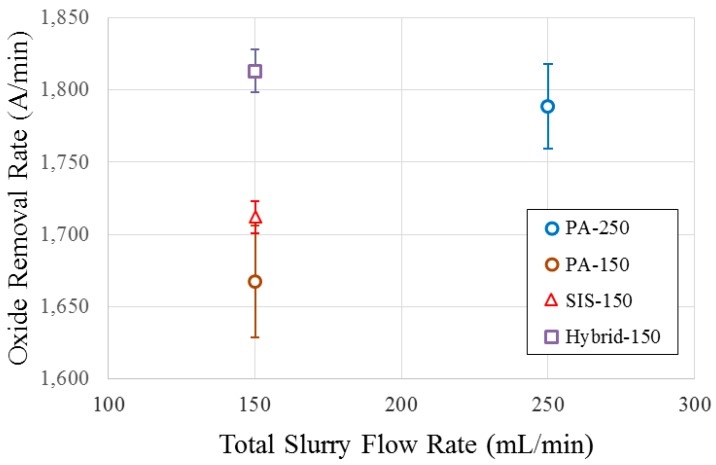
Summary of removal rate data. PA-250: polishing using the PA method at a slurry flow rate of 250 mL/min; PA-150: polishing using the PA method at a slurry flow rate of 150 mL/min; SIS: polishing using the SIS method at a slurry flow rate of 150 mL/min; Hybrid-150: polishing using the hybrid method (SIS at a slurry flow rate of 50 mL/min + PA at a slurry flow rate of 100 mL/min) at a total slurry flow rate of 150 mL/min.

Several published reports indicated that 300-mm CMP processes typically employ slurry flow rates ranging from 250 mL/min to 300 mL/min to achieve optimum material removal rates (RR) [11,23,24,25]. In a separate study, Wang et al. showed that RR increases with the PA slurry flow rate, but it eventually reaches an asymptote where further increases in slurry flow rate no longer affect removal rate [26]. Philipossian et al. showed that the slurry utilization efficiency (defined as the portion of slurry that flows through the pad–wafer interface, divided by the total amount of slurry applied) actually decreases with an increase in slurry flow rate [8]. Simply increasing slurry flow rate until a certain level may help achieve higher RR in several cases, but at a disproportionality higher consumable cost. Therefore, in this study, polishing using the PA method at a slurry flow rate of 250 mL/min (henceforth referred to as “PA-250”) is considered as our baseline process. Figure 3 summarizes the removal rates for all slurry injection methods (to be elaborated in detail later on in this section). As expected, PA at reduced slurry flow rate (i.e., 150 mL/min, henceforth referred to as “PA-150”) yields lower RR than PA-250. Several published results have reported a similar finding, whereby removal rates are shown to decrease with slurry flow rate [11,26]. One reason for this observation is that at the reduced flow rate, less slurry is available to be transported to the pad–wafer interface [21,27]. Another reason is the degree of slurry mixing: as discussed in Section 1, during polishing, the fluid residing on the pad surface contains spent slurry, polishing by-products (i.e., pad debris and chemical by-products), and residual rinse water from the pad rinsing procedure performed between polishes which do not contribute to RR [11,27]. The fresh slurry injected on top of the pad surface gets diluted by mixing with spent slurry and residual rinse water. The net effect is the reduction of the fraction of fresh slurry delivered to the polishing region (i.e., between the pad and the wafer) where the removal mechanism occurs. At reduced slurry flow rate (i.e., the PA-150), the fraction of fresh slurry becomes even lower, thus leading to a lower RR [11,17,27]. As a matter of fact, the traditional PA method does not provide a mechanism to promote a higher fraction of fresh slurry. Based on the explanation above, we can infer that both the degree of slurry mixing and slurry availability affect RR during polishing processes.

**Figure 4:**
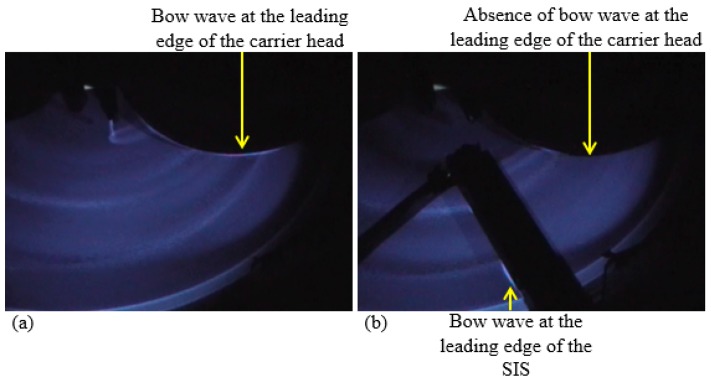
Bow wave formation on the polishing pad using (**a**) Point Application (PA) method and (**b**) the SIS.

Previous studies showed that slurry mean residence time (MRT) of a certain CMP process is an indicator for the degree of slurry mixing, such that a higher value of MRT means more mixing between the freshly injected slurry and spent slurry, as well as residual rinse water [11,17]. Therefore, lower values of MRT are desirable in CMP because more fresh slurry is being delivered to the pad–wafer interface at a faster rate. Meled et al. and Mu et al. have shown that SIS significantly decreases slurry MRT compared to the PA method [11,17]. Furthermore, Mu et al. concluded that the dispersion number is lower with SIS, which accounts for the lower MRT [17]. The mechanism can be further explained with the UVEF images shown in Figure 4. With the PA injection scheme, a thick bow wave containing spent slurry and residual rinse water is formed directly at the leading edge of the wafer carrier head, as shown in Figure 4a. Consequently, the PA method allows more spent slurry and residual rinse water to re-enter the wafer–pad interface. In contrast, as the SIS is placed in front of the leading edge of the wafer carrier head, it prevents spent slurry and residual rinse water from re-entering the pad–wafer interface during polishing. Figure 4b shows the UVEF image of a polishing process using an SIS design that covers only the outer half of the wafer track. Similar to a regular SIS design (i.e., covering the whole wafer track), the bow wave was formed at the leading edge of the SIS during polishing. Due to the centrifugal force of the platen and wafer rotation, most of the polishing by-products, spent slurry, and residual rinse water dominantly reside closer to the edge of the pad rather than to the center of the pad. Therefore, having a smaller SIS design that covers only the outer half of the pad is still desirable. As shown in Figure 4b, an SIS design that covers the outer half of the wafer track can effectively block polishing by-products, spent slurry, and residual rinse water from re-entering the pad–wafer interface. The thick bow wave formed at the leading edge of the SIS closer to the edge of the pad confirms that the spent slurry and residual rinse water are effectively blocked from re-entering the pad–wafer interface and guided off from the pad surface to the drainage. Furthermore, by using the SIS, the fresh slurry is less diluted with the spent slurry and residual rinse water, leading to a higher fraction of fresh slurry that enters the pad–wafer interface.

**Figure 5:**
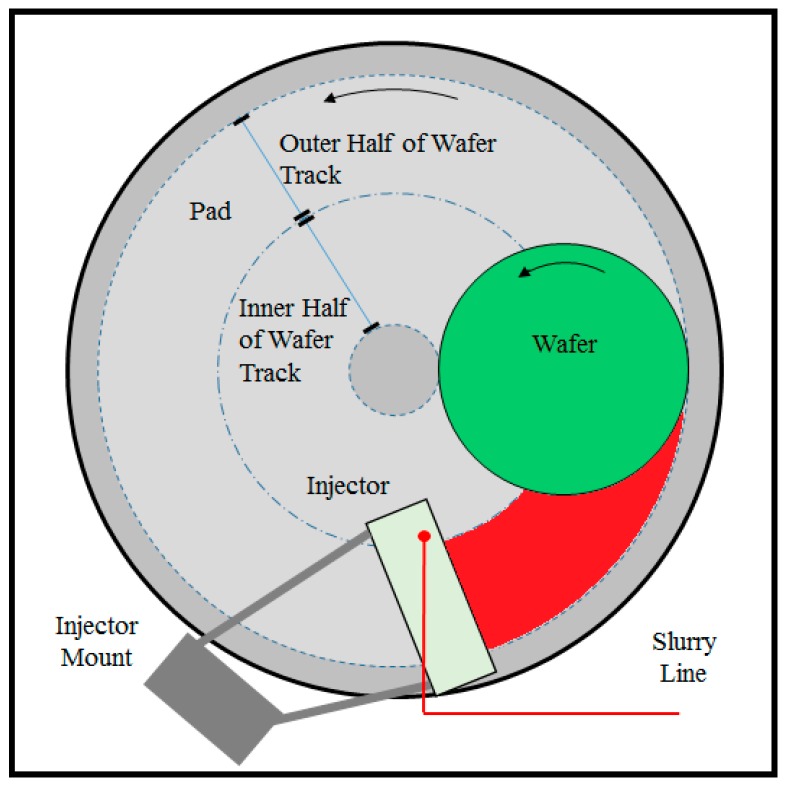
Coverage of fresh slurry on wafer track (red) using slurry injection system with slurry injection point coinciding with the center of the wafer track.

**Figure 6:**
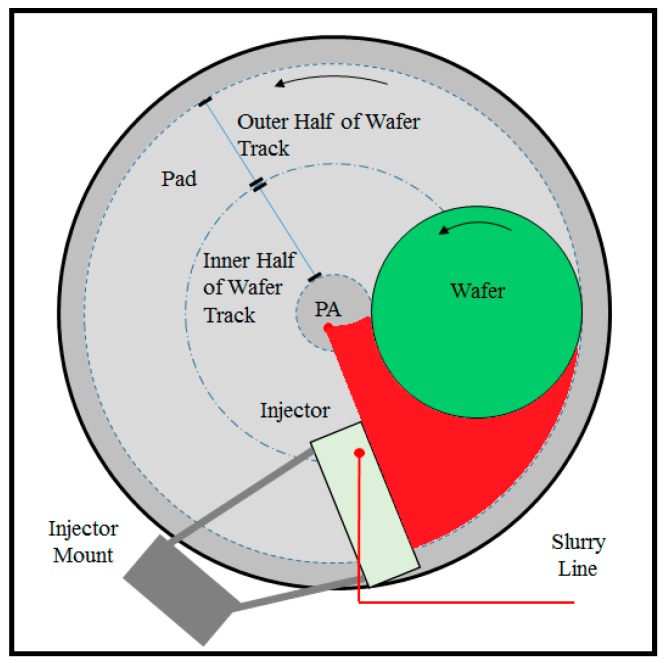
Coverage of fresh slurry on wafer track (red) using a hybrid system.

SIS increases the slurry availability and reduces the degree of mixing of the fresh slurry with the spent slurry and residual rinse water. As a result, using the SIS at a reduced slurry flow rate can achieve similar removal rates compared to PA [11,17,21]. Previous studies on the SIS (that fully cover the whole wafer track) showed that SIS at a slurry flow rate of 150 mL/min achieved the same removal rate as PA at a slurry flow rate of 250 mL/min [11,17,21]. Referring back to Figure 3, using an SIS that only covers the outer half of the wafer track at a flow rate of 150 mL/min (referred to as “SIS-150”) yields an RR that is significantly lower than the PA-250 and only slightly higher than the PA-150. In this configuration, the slurry injection port on the SIS coincides with the center of the wafer track. Due to rotation and centrifugal forces of both platen and wafer, the injected fresh slurry is then mainly distributed on the outer half of the wafer track, as illustrated by red color in Figure 5. Therefore, injecting the slurry in the center of the wafer track reduces slurry coverage in the pad–wafer interface, as entering fresh slurry is now reduced to one-half of what was observed when PA and full SIS designs were used. Furthermore, centrifugal forces act more rapidly to pull the slurry off of the pad surface as the fresh slurry in injected closer to the edge of the pad. Therefore, in this configuration, while SIS is still effective in blocking the spent slurry and residual rinse water, slurry availability is greatly reduced because it only covers the outer half of the wafer track. To increase slurry availability on the inner half of the wafer track while still taking the benefit of SIS to block the spent slurry and residual rinse water to re-enter the pad–wafer interface, a hybrid system is proposed as shown in Figure 6. 

Figure 6 illustrates the hybrid system that combines both SIS and PA (what we will henceforth refer to as “Hybrid”). In this case, fresh slurry streams are injected contemporaneously to the SIS as well as to point application (PA) at 50 and 100 mL/min, respectively. The total slurry flow rate remains the same at 150 mL/min (i.e., 40 percent reduction from the 250 mL/min using the regular PA method). The main purpose for having slurry injected through the PA is to facilitate the availability of fresh slurry in the inner half of the wafer track. In the inner half of the wafer track, slurry dilution (with spent slurry and residual rinse water) is unavoidable, since SIS does not cover this region. However, the dilution is expected to be significantly less pronounced than the outer half of the wafer track. Similarly, the location of the SIS is kept constant (i.e., covering the outer half of the wafer track) in order to effectively block the spent slurry and residual rinse water from re-entering the pad–wafer interface as previously explained. At the same time, slurry is injected via SIS to ensure enough availability of fresh slurry on the outer half of the wafer track. 

In summary, the hybrid system is expected to provide enough availability of fresh slurry covering the whole wafer track and to still cause the removal of spent slurry and residual rinse water. As shown in Figure 3, the Hybrid-150 yields an RR that is comparable to or slightly higher than the PA-250. Compared to PA, the hybrid system accommodates the delivery of fresh slurry, as it covers the whole wafer track and increases the fraction of fresh slurry delivered to the pad–wafer interface (i.e., polishing region where the removal mechanism occurs) by squeegeeing (i.e., wiping) off most of the spent slurry and residual rinse water. 

**Figure 7:**
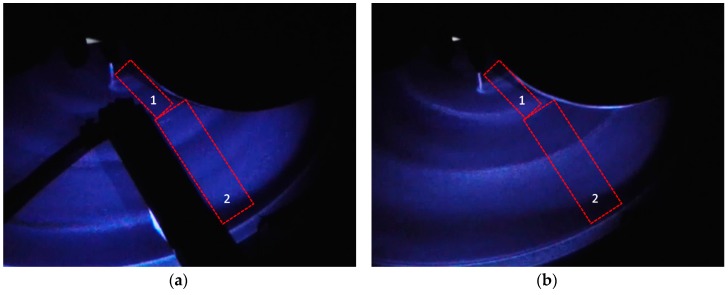
Regions 1 and 2 for UVEF analysis on the polishing pad: (**a**) with the SIS, and (**b**) without the SIS.

To further explain the above results, a UVEF technique was employed to qualitatively measure slurry availability on top of the pad surface. Table 1 summarizes the average UVEF intensity throughout the polishing time, obtained by analyzing UV-enhanced fluid film on the two regions depicted in Figure 7. Regions 1 and 2 are located on the inner and outer half of the wafer track, respectively. Both regions are located on the pad prior to the entering of the slurry in the pad–wafer interface. With the SIS installed, both regions are intentionally set between the SIS and wafer carrier head. For a fair comparison among PA, SIS, and hybrid systems, the location for both Regions 1 and 2 was kept the same. It is important to note that UVEF intensity in this study does not measure an exact fluid film volume, but rather a relative slurry availability based on findings in a previous study [21]. The UVEF intensity increases with slurry availability and vice versa. In addition, such a technique is not intended to exactly quantify the composition of the fluid film (i.e., spent slurry, fresh slurry, and residual rinse water). As shown in Table 1, PA-250 results in the highest UVEF intensity measured in both Regions 1 and 2. This is intuitive because this particular slurry application method uses the highest flow rate and has no mechanism for squeegeeing off the spent slurry. 

Compared to PA-250, PA-150 decreases the UVEF image intensity by approximately 9% and 14% in Regions 1 and 2, respectively. Such decreases are expected due to reductions in the injection of fresh slurry. It must be noted that a controlled pad rinsing procedure with UPW (that contains no fluorescence dye) is performed prior to every polish. As such, the amount of residual rinse water on the pad is the same prior to every polish on both the PA-250 and PA-150. At a reduced slurry flow rate (i.e., PA-150), the freshly injected slurry is diluted more with the residual rinse water. Furthermore, a lower slurry flow rate takes a longer time to replace the residual rinse water with the dyed slurry. Since the mechanisms of slurry injection and fluid removal are essentially the same between PA-250 and PA-150, this reduction in image intensity associated with PA-150 confirms a lower fraction of fresh slurry to overall fluid on the pad during polishing compared to PA-250. As a result, the RR of PA-150 is lower than that of PA-250.

Figure 3 shows that the RR of the SIS-150 is marginally higher than PA-150. In the meantime, Table 1 shows that when changing the slurry application mechanism from PA-150 to SIS-150, the UVEF image intensity decreases significantly by approximately 45% and 27% in Regions 1 and 2, respectively. Compared to PA, SIS incorporates a mechanism that squeegees and wipes off the residual rinse water as well as the spent slurry that contains the dye. During polishing, the spent slurry has a reduced polishing capability (in terms of RR) compared to fresh slurry, but the dye component itself is not degraded in terms of fluorescence intensity. By effectively squeegeeing off the spent slurry, SIS-150 is artificially showing less slurry availability compared to PA-150 as shown by its lower UVEF image intensity. In fact, SIS prevents the spent slurry from re-entering the pad–wafer region, and therefore the UVEF image intensity associated with SIS in Regions 1 and 2 can be regarded as a much higher fraction of the fresh slurry (i.e., less dilution with spent slurry). Compared to PA-150, the significant drop of UVEF intensity with SIS-150 in Region 1 is attributed to the absence of slurry injection in the inner half of the wafer track. As a result, only spent slurry contributes to the UVEF intensity with the SIS-150 in Region 1. Region 2 of the SIS-150 has a significantly higher intensity than Region 1 because the fresh slurry is injected through the SIS that covers the outer half of the wafer track (i.e., Region 2). The UVEF technique shows that even though SIS-150 can effectively wipe off the spent slurry and residual rinse water, the fresh slurry is mainly available only to the outer half of the wafer track.

Compared to SIS-150, the Hybrid-150 increases the UVEF intensity of Regions 1 and 2 by approximately 40% and 30%, respectively. In Region 1, the sharp increase in intensity is attributed to the addition of slurry injection on the inner half of the wafer track. As a result, it increases the fraction of fresh slurry to the overall fluid and hence the slurry availability in the inner half of the wafer track. The increase of intensity in Region 2 also outlines the effect of full wafer track slurry coverage, as illustrated in Figure 6. The rotation of the platen–wafer ejects the slurry from the inner half of the wafer track toward the outer half of the wafer track. Such a mechanism, combined with the fresh slurry injected through the injector, increases slurry availability in Region 2 and thereby causes higher UVEF intensity. Using the hybrid method, the combined mechanisms of the full slurry coverage and the squeegee effects (of spent slurry and residual rinse water) increase the fraction of fresh slurry while maintaining slurry availability on top of the polishing pad during polishing.

## 4. Conclusions

This work has shown that slurry availability and the extent of slurry mixing (i.e., among fresh slurry, spent slurry, and residual rinse water) dramatically influence removal rates. The ultraviolet enhanced fluorescence (UVEF) technique showed that injecting fresh slurry solely on the center of wafer track reduces slurry availability in the pad–wafer interface, as it only covered the outer half of the wafer track. Removal rates were found to decrease with slurry availability accordingly. A higher degree of slurry mixing decreased the fraction of fresh slurry, and consequently lowered the removal rate. In this study, a novel slurry injection system was installed on top of the polishing pad that covered only the outer half of the wafer track. UVEF technique confirmed that most of the fluid (i.e., slurry and residual rinse water) predominantly resided closer to the edge of the pad rather than near the center of the pad. Therefore, the SIS that covered only the outer half of the wafer track was still effective in blocking the spent slurry and the residual rinse water. This mechanism facilitated the increase in the fraction of fresh slurry on the polishing pad during polishing. In contrast, standard pad center area slurry application did not have a similar mechanism to block the spent slurry and residual rinse water.

Results further indicated that slurry injection position and the novel SIS played important roles in slurry mixing characteristics and slurry availability on top of the pad. Injecting fresh slurry at a higher flow rate and at a location closer to the pad center area generally increased slurry availability. By using a hybrid system (i.e., a combination of slurry injection through the shorter SIS and a standard pad center area slurry application), the polishing process benefited from higher slurry availability and higher fraction of fresh slurry than the pad center area slurry application and the shorter SIS, individually. This was verified by the UVEF technique.

## Figures and Tables

**Table 1:** Summary of ultraviolet enhanced fluorescence (UVEF) images analysis.

**Region 1**		
**Slurry Injection Scheme**	**Average UVEF Intensity (A.U.)**	**Standard Deviation (A.U.)**
PA-250	53.5	1.9
PA-150	48.8	3.3
SIS-150	27.0	1.2
Hybrid-150	44.7	2.5
**Region 2**		
**Slurry Injection Scheme**	**Average UVEF Intensity (A.U.)**	**Standard Deviation (A.U.)**
PA-250	66.0	3.5
PA-150	57.1	3.4
SIS-150	41.8	2.4
Hybrid-150	59.9	2.2
